# Leadless Pacemaker Implantation in Fabry Disease With Permanent Atrial Fibrillation and Bradycardia: Diagnostic and Implantation Guidance by Preprocedural Cardiac Magnetic Resonance Imaging

**DOI:** 10.1002/ccr3.73917

**Published:** 2026-11-09

**Authors:** Hiroki Saito, Yuya Suzuki, Shinichiro Yamada, Hideki Fujii, Kaoru Takami, Yu Takahashi, Kimi Nishijyo, Akihiro Yoshida

**Affiliations:** ^1^ Division of Cardiology Kita‐Harima Medical Center Ono Japan; ^2^ Division of Nephrology Kobe University Graduate School of Medicine Kobe Japan

**Keywords:** atrial fibrillation, atrioventricular block, cardiac magnetic resonance imaging, Fabry disease, late gadolinium enhancement, leadless pacemaker

## Abstract

In advanced Fabry cardiomyopathy, preprocedural cardiac magnetic resonance imaging may reveal a lower septal region spared from otherwise extensive septal late gadolinium enhancement. This information, combined with pre‐fixation electrical mapping, may help identify an optimal site for leadless pacemaker implantation and minimize repeated fixation attempts.

## 1 Introduction

Fabry disease is an X‐linked lysosomal storage disorder resulting from an α‐galactosidase A deficiency, leading to the systemic accumulation of globotriaosylceramide (Gb3) and subsequent multiorgan involvement. Cardiac manifestations are common and include progressive left and right ventricular (RV) hypertrophy, conduction system disease, and arrhythmias such as atrial fibrillation (AF) and bradyarrhythmia (sinus node dysfunction and atrioventricular block), often requiring pacemaker implantation [1, 2].

This case report describes leadless pacemaker (LP) implantation in a patient with Fabry disease and bradycardia, highlighting procedural considerations in biventricular hypertrophy and atrial remodeling.

## 2 Case History and Examination

A 76‐year‐old male with a history of permanent AF, chronic heart failure, and hypertension presented with symptomatic bradycardia and exertional dyspnea. Approximately 1 month before admission, the patient developed exertional dyspnea that gradually worsened and was accompanied by bilateral lower leg edema. At admission, his medications included warfarin and diuretics.

Electrocardiography demonstrated AF with bradycardia and a heart rate of 35 beats/min. Chest radiography showed signs of congestive heart failure. Echocardiography revealed a marked biatrial enlargement and ventricular hypertrophy (Figure 1). The left ventricular (LV) ejection fraction was preserved at 61% and no significant valvular disease was observed. Laboratory evaluation showed elevated levels of high‐sensitivity troponin I and B‐type natriuretic peptide (BNP), while renal function was preserved, and no proteinuria was detected.

**FIGURE 1:**
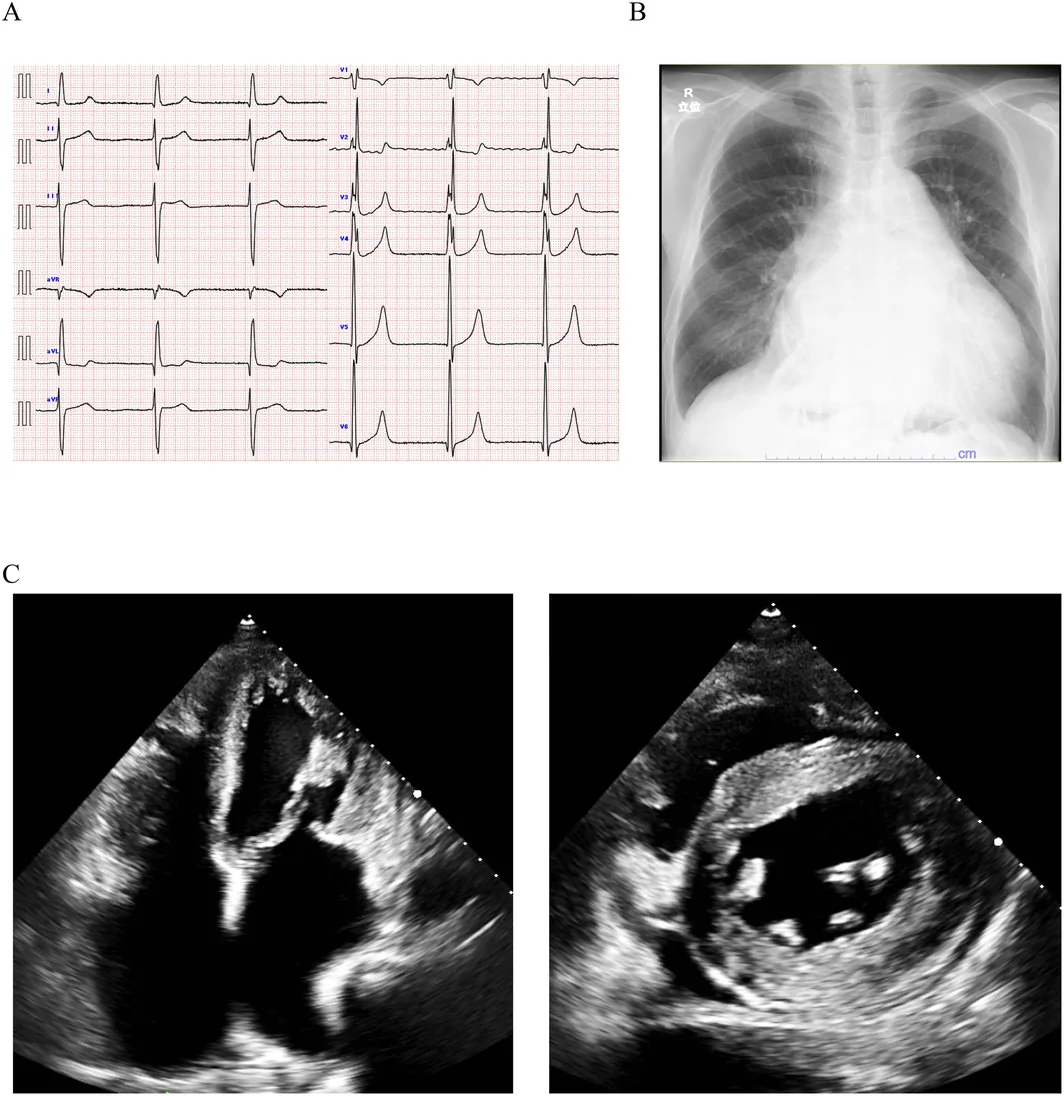
Twelve‐lead ECG, chest X‐ray, and echocardiography. (A) A 12‐lead ECG showing atrial fibrillation with bradycardia. (B) Chest X‐ray demonstrating cardiomegaly and mild bilateral pleural effusion. (C) Transthoracic echocardiography revealing biatrial enlargement and left and right ventricular hypertrophy.

## 3 Differential Diagnosis, Investigations, and Treatment

In patients presenting with unexplained ventricular hypertrophy, key differential diagnoses include sarcomeric hypertrophic cardiomyopathy, cardiac amyloidosis, Fabry disease, and hemochromatosis. Cardiac magnetic resonance imaging (CMR) revealed globally decreased native T1 values along with diffuse late gadolinium enhancement (LGE) involving the interventricular septum; however, no LGE was observed in the inferior portion of the septum (Figure 2). A global reduction in native T1 values is a rare finding and substantially narrows the differential diagnosis, most notably to Fabry disease or hemochromatosis. However, hemochromatosis was excluded based on normal iron metabolism markers (normal serum ferritin and transferrin saturation) and normal magnetic resonance imaging (MRI) T2 values, which typically decrease in cases of iron deposition.

**FIGURE 2:**
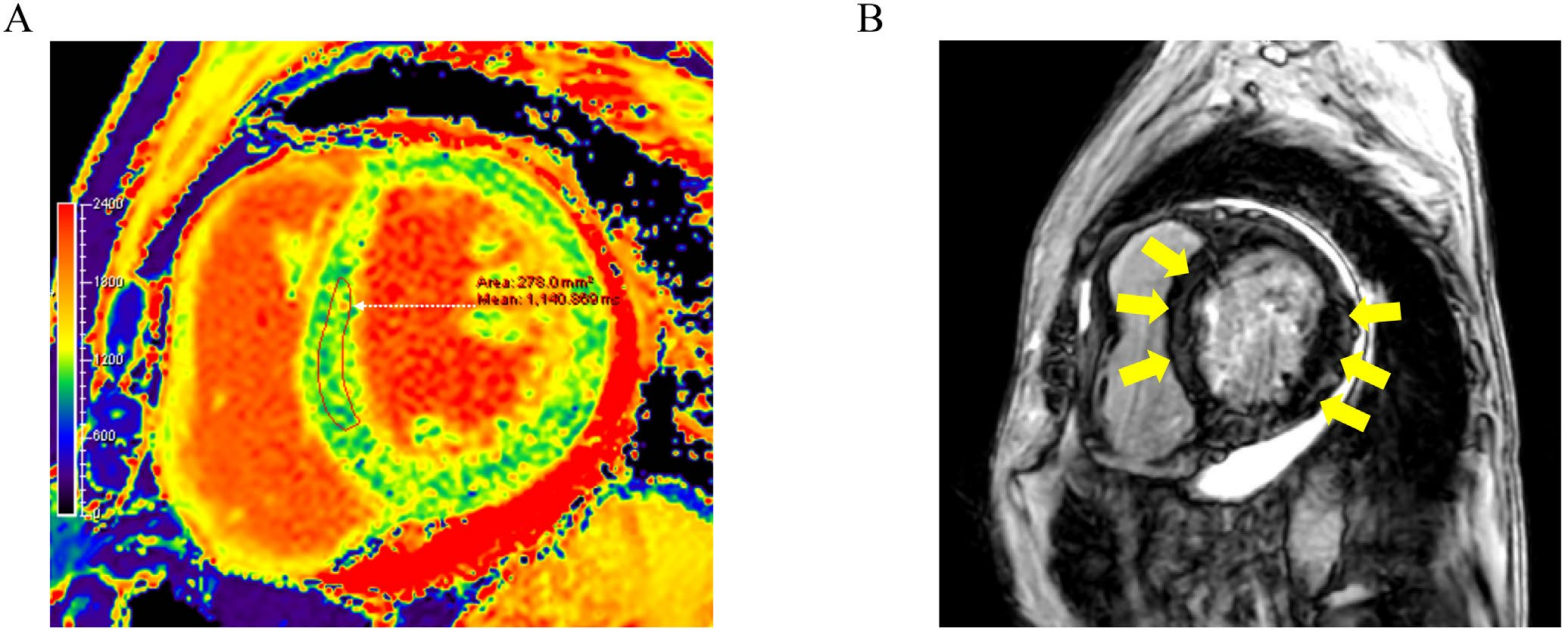
Cardiac magnetic resonance imaging (CMR). (A) Native T1 mapping showing diffusely reduced T1 values; the interventricular septal T1 was 1140 ms (institutional normal range: 1256 ± 25 ms). (B) Late gadolinium enhancement (LGE) image revealing diffuse enhancement of the left ventricle, with the interventricular septum showing enhancement except for the inferior portion.

These findings, combined with RV myocardial biopsy showing vacuolated myocytes, absence of iron deposition on Berlin blue staining, and the presence of concentric, electron‐dense inclusions within myofibrils on electron microscopy, were suggestive of Fabry disease (Figure 3). The diagnosis of Fabry disease was established based on decreased α‐galactosidase A activity (6.8 nmol/mg) together with characteristic findings on cardiac magnetic resonance imaging and endomyocardial biopsy. Furthermore, genetic analysis identified an intron 4 variant in the GLA gene (c.640‐801G>A). The overall clinical presentation was most consistent with a late‐onset phenotype.

**FIGURE 3:**
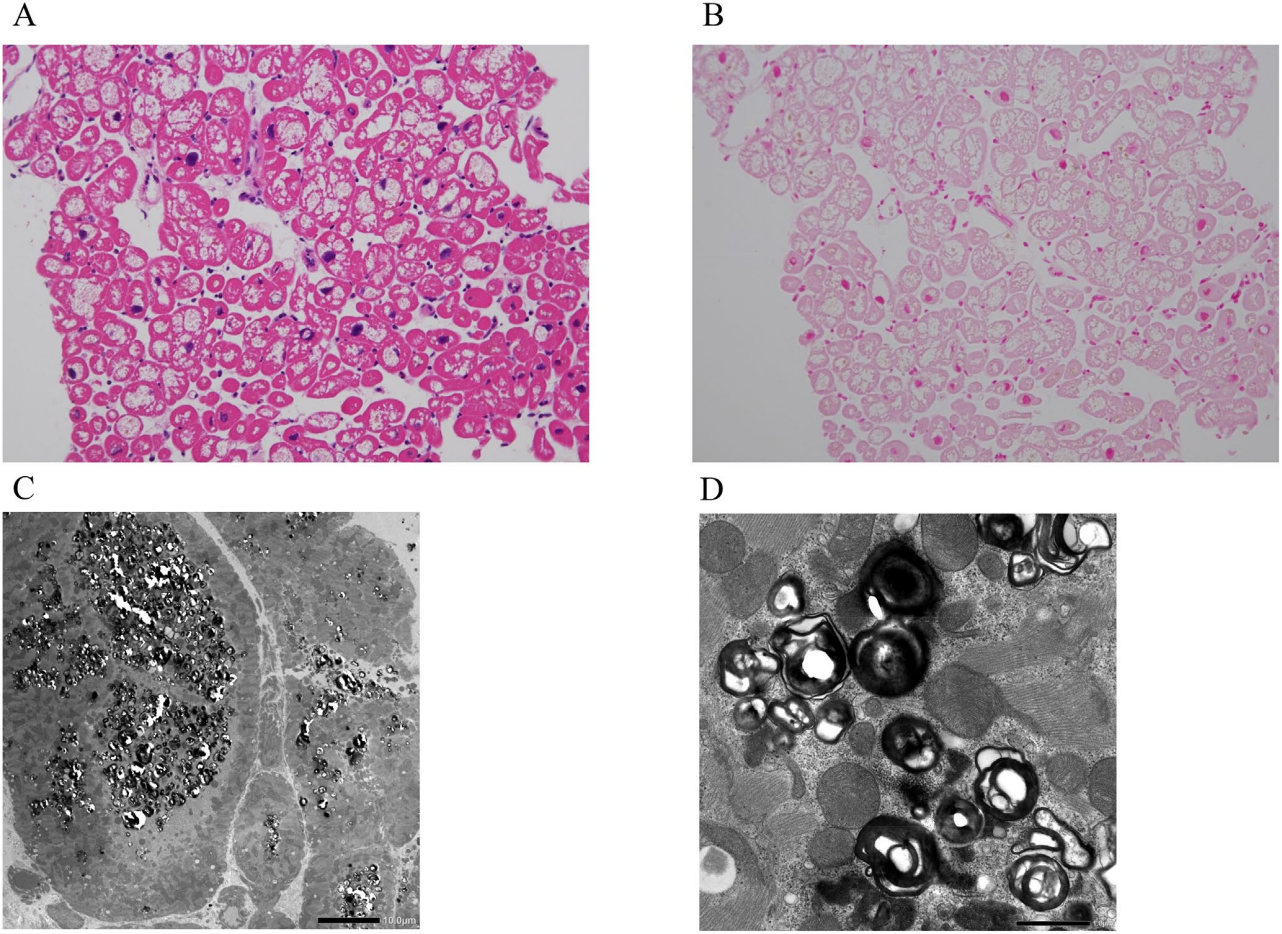
Myocardial pathology. (A) Hematoxylin and eosin (H&E) staining (×400) of the myocardial tissue showing prominent cytoplasmic vacuolization of cardiomyocytes. (B) Berlin blue stain (×400). It is negative for iron deposition. (C, D) Electron microscopy showing concentric electron‐dense inclusions within the myofibrillar structures.

Given the patient's permanent AF and symptomatic bradycardia, permanent pacing was indicated. Permanent AF made single‐chamber ventricular pacing appropriate, and the preimplantation LVEF was preserved at 61%. Cardiac resynchronization therapy (CRT) was not selected as the initial pacing strategy. Septal LGE raised concerns regarding the technical feasibility of left bundle branch area pacing (LBBAP). Extensive septal scar has been associated with difficulty deploying a lead to the LV septum via a transseptal approach [3]. These considerations, together with the patient's preference for a leadless system, informed the selection of leadless pacing over a transvenous system. An implantable cardioverter‐defibrillator (ICD) was not selected because the patient had preserved LV systolic function and had experienced neither sustained ventricular arrhythmias nor nonsustained ventricular tachycardia. Therefore, pacing therapy alone was chosen after an individualized risk assessment. Considering the progressive nature of Fabry disease, however, the possibility of future LV dysfunction or ventricular arrhythmias requiring additional therapy, such as cardiac resynchronization therapy with defibrillation (CRT‐D), cannot be excluded. Therefore, a leadless pacemaker with an active‐fixation helix (Aveir VR, Abbott, Chicago, IL, USA) was selected over the Micra VR device because of its potential for future retrieval. Moreover, LP implantation was expected to be challenging due to marked atrial enlargement and septal late gadolinium enhancement on CMR. Unlike the Micra VR device, Aveir VR allows pre‐fixation mapping before fixation. Therefore, Aveir VR was selected to use pre‐fixation mapping to identify an optimal target site while minimizing the number of LP deployments/screw‐in attempts, thereby potentially reducing myocardial injury and procedural risk.

Right ventricular angiography was performed during the implantation procedure (Figure 4A). During pre‐fixation mapping at the high and midseptal sites (Figure 4B,C), ventricular capture could not be achieved at 5.0 V with a pulse width of 0.4 ms. Exact pacing thresholds, R‐wave amplitudes, and impedance values at these sites could not be retrieved from the available records. In contrast, favorable electrical parameters were obtained at the inferior septal site (Figure 4D), with a pacing threshold of 0.75 V at 0.4 ms, an R‐wave amplitude of 6.0 mV, and an impedance of 610 Ω. After fixation at this site, the pacing threshold was 0.5 V at 0.4 ms, the R‐wave amplitude was 7.2 mV, and the impedance was 700 Ω. CMR revealed an LGE‐negative area limited to the inferior interventricular septum, whereas LGE was present in the remaining septal segments. The anatomical concordance between this LGE‐negative region and favorable capture during pre‐fixation mapping suggests that preprocedural CMR may help identify a suitable implantation site. Successful implantation was achieved with a single screw‐in maneuver.

**FIGURE 4:**
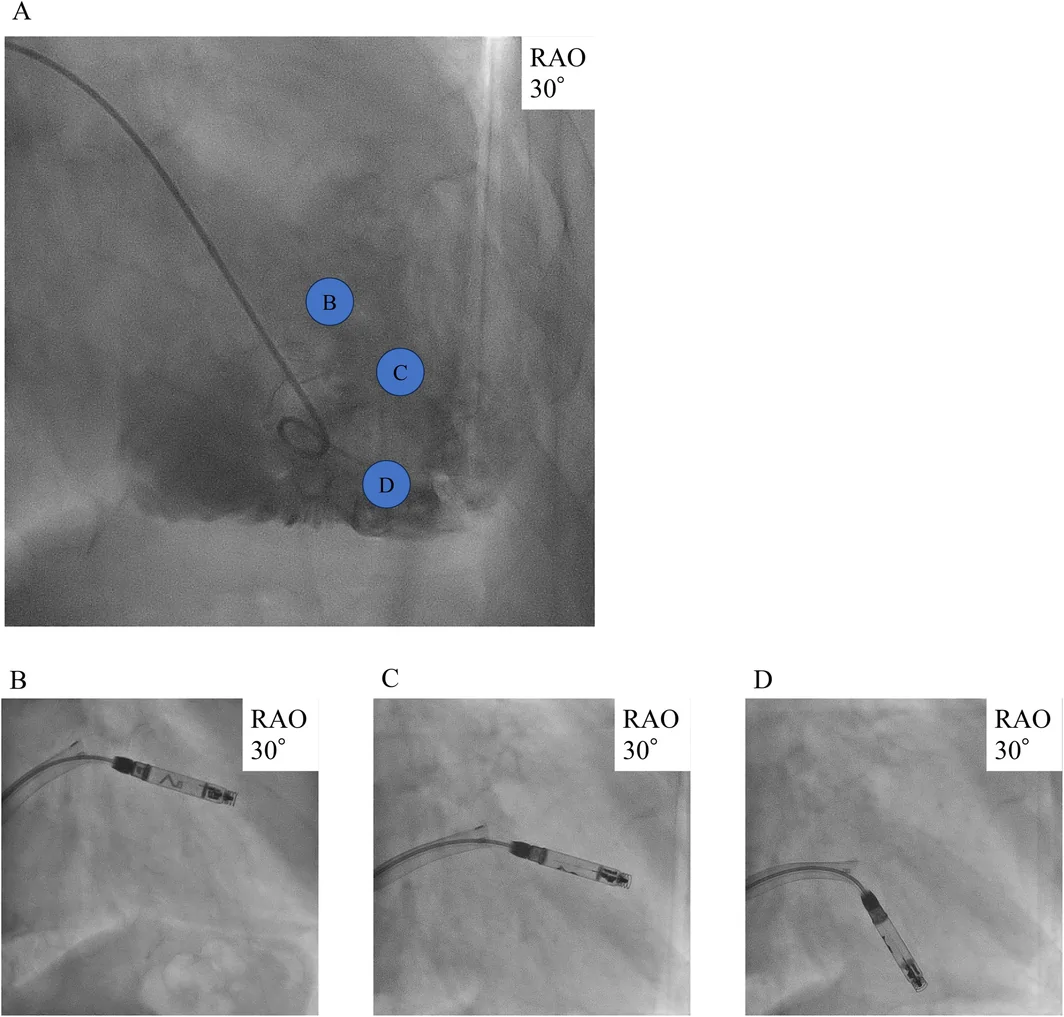
Intraoperative fluoroscopic images during Aveir VR implantation. (A) Right ventricular angiography. (B–D) The sites evaluated during pre‐fixation mapping. (B) Pre‐fixation mapping at the high septal site: Ventricular capture could not be achieved at 5.0 V with a pulse width of 0.4 ms. (C) Pre‐fixation mapping at the midseptal site: Ventricular capture could not be achieved at 5.0 V with a pulse width of 0.4 ms. Exact thresholds, R‐wave amplitudes, and impedance values at the sites in B and C were unavailable. (D) Final implantation site in the inferior septum, where favorable electrical parameters were confirmed during pre‐fixation mapping, with a pacing threshold of 0.75 V at 0.4 ms, an R‐wave amplitude of 6.0 mV, and an impedance of 610 Ω. After fixation, the pacing threshold was 0.5 V at 0.4 ms, the R‐wave amplitude was 7.2 mV, and the impedance was 700 Ω.

## 4 Outcome and Follow‐Up

The LP remained stable with satisfactory pacing thresholds. No periprocedural or late device‐related complications, including cardiac perforation or tamponade, were observed during the 25‐month follow‐up period, and no heart failure symptoms or ventricular arrhythmias were reported during that period. Despite the progressive nature of Fabry cardiomyopathy and the potential indication for enzyme replacement therapy, the patient strongly preferred not to receive disease‐specific treatment and wished to receive only symptom‐directed management, including treatment of heart failure. Management therefore consisted of symptom‐directed treatment and continued clinical follow‐up. Preimplantation TTE performed during complete atrioventricular block showed an LVEF of 61%. LVEF was 46% approximately 2 months after implantation and 45% approximately 20 months after implantation, indicating no further decline between the two postimplantation assessments. Device interrogation approximately 25 months after implantation showed a right ventricular pacing percentage of 71%. These measurements were obtained at separate follow‐up visits.

## 5 Discussion

To the best of our knowledge, this is the first reported case of leadless pacemaker implantation in a patient with Fabry disease. This case illustrates the potential role of preprocedural CMR combined with pre‐fixation mapping in selecting a suitable site for LP implantation in Fabry cardiomyopathy.

Several studies have reported that the prevalence of Fabry disease among patients with unexplained LV hypertrophy is approximately 1%–3% [4]. Therefore, it is of utmost importance to consider Fabry disease in all cases of HCM and to perform careful exclusionary diagnostics. In the present case, preprocedural CMR imaging demonstrated diffuse shortening of native T1 values, which played a pivotal role in the diagnostic process. Among conditions characterized by low native T1 values [5], Fabry disease and hemochromatosis were considered. However, hemochromatosis was deemed unlikely, given the absence of a transfusion history and normal serum iron and ferritin levels. Consequently, Fabry disease emerged as the leading differential diagnosis, prompting further evaluation, including measurement of α‐galactosidase A activity and endomyocardial biopsy, which ultimately confirmed the diagnosis. Although Fabry disease is a rare disorder, effective disease‐specific therapies are available, making early diagnosis and timely initiation of treatment highly important. Of particular concern is the irreversible involvement of the cardiac conduction system; therefore, the benefits of early detection and intervention are substantial. Moreover, enzyme replacement therapy is more effective when introduced before the onset of myocardial fibrosis, further emphasizing the necessity of early recognition and treatment [6]. In the present case, left ventricular hypertrophy had been documented 20 years before the current hospitalization for heart failure. If a thorough evaluation of hypertrophy had been performed at that time, treatment could have been initiated before the development of conduction disturbances, potentially halting disease progression.

The type of genetic variant largely determines the clinical phenotype. Frameshift variants such as deletions or duplications, nonsense variants, and some missense or splicing variants result in absent or markedly reduced α‐galactosidase A activity, leading to the classic phenotype. In contrast, other missense and splicing variants are associated with residual enzyme activity and give rise to a late‐onset phenotype, which predominantly manifests as cardiac involvement and, less commonly, renal disease [7].

The GLA c.640‐801G>A variant (NM_000169.3), also known as IVS4+919G>A, identified in this patient is a pathogenic deep intronic variant associated with late‐onset, cardiac‐predominant Fabry disease. It promotes inclusion of a 57‐nucleotide intronic sequence in the transcript, resulting in aberrant splicing and reduced α‐galactosidase A activity [8]. In this patient, the onset of clinical manifestations was presumed to occur in his fifties, when LV hypertrophy was first noted, which is consistent with this phenotype.

Enzyme replacement therapy warranted consideration in this patient with established Fabry cardiomyopathy. However, disease‐specific treatment was not initiated because of the patient's strong preference for symptom‐directed management rather than a lack of potential treatment indication.

Fabry disease frequently affects the cardiac conduction system, with progressive infiltration and hypertrophy involving both the ventricles and atria [9, 10]. RV hypertrophy, although less frequently highlighted than LV involvement, is common and may contribute to procedural challenges during device implantation [10].

The need for pacemaker implantation in patients with Fabry disease has been well documented, with conduction disturbances and bradyarrhythmias reported in 10%–20% of cases [11, 12]. In patients with the late‐onset phenotype of Fabry disease, complete atrioventricular block has been reported in 5.9% overall, including 12.7% of males and 1.6% of females [6]. Although severe bradyarrhythmias are relatively common in patients with Fabry‐associated cardiomyopathy, they rarely present early in the disease course. Instead, they typically occur at a later stage and are consistently associated with advanced cardiac involvement. However, due to the rarity of this disease, reports on device implantation remain limited. A recent study highlighted conduction disturbances and device therapy in the Fabry cohort; however, no cases of LP implantation were included [13].

Among the currently available LP systems, the Aveir VR system offers several advantages for structurally complex heart disease. Compared to Micra VR and Micra AV (Medtronic, Minneapolis, MN, USA), which use passive‐fixation tines, Aveir VR provides a longer estimated battery life, the ability to perform pre‐fixation mapping of the electrical local signal prior to device fixation, and the option for future device retrieval. Aveir VR uses an active‐fixation helix that screws into the RV endocardium and has a dedicated retrieval kit from the manufacturer. For the predecessor Nanostim leadless pacemaker, a chronic retrieval success rate of 87.6% has been reported, with implant durations of up to 9 years [14]. These findings provide supporting evidence for the retrieval concept, although they do not directly establish long‐term retrieval outcomes for Aveir VR. These features are especially relevant in patients with infiltrative cardiomyopathies and extensive chamber hypertrophy, where securing an optimal implant site can be technically challenging. While Micra AV offers synchronous pacing via accelerometer‐based sensing [15], this was not necessary in the patient because of permanent AF. In this case, pre‐fixation mapping enabled the identification of high‐threshold zones in the RV septum, allowing the operator to avoid multiple deployment attempts, thereby minimizing myocardial trauma. Furthermore, the potential for progressive cardiomyopathy in Fabry disease, with a possible future need for CRT, favors the use of Aveir VR.

Procedural guidance for implantation of a leadless pacemaker with an active‐fixation helix describes septal site selection [16]. In this case, pre‐fixation mapping demonstrated failure to capture at 5.0 V at the high and midseptal sites, whereas favorable capture was observed at the inferior septal site. CMR revealed LGE in the interventricular septum except in the inferior portion, which showed no LGE. The inferior septal region was therefore selected for implantation. Ali et al. reported that extensive septal scar was associated with difficulty deploying a lead to the LV septum via a transseptal approach during LBBAP [3]. Although this evidence concerns LBBAP rather than LP implantation, it supports considering septal scar distribution when planning a pacing procedure. In the present case, the anatomical concordance between the LGE‐negative inferior septal region and favorable capture during pre‐fixation mapping suggests that CMR may help guide site selection.

The preimplantation LVEF was measured during complete atrioventricular block, and differences in heart rate and loading conditions may have contributed to the difference between preimplantation and postimplantation measurements. However, a contribution from RV pacing to the early reduction in LVEF cannot be excluded. Although LVEF remained similar at 2 and 20 months after implantation, continued assessment of ventricular function and heart failure status is warranted given the substantial RV pacing burden and underlying Fabry cardiomyopathy. The potential need for CRT or another physiologic pacing strategy should be reassessed according to the subsequent clinical course. This case highlights the importance of individualized device selection and implantation strategies in patients with rare cardiomyopathies.

## 6 Conclusions

This report describes a case of leadless pacemaker implantation in a patient with Fabry disease complicated by marked biatrial dilation and extensive septal involvement, sparing only the most inferior portion.

Procedural success highlights key considerations, including determination of the implantation site based on LGE findings and appropriate selection of the leadless pacemaker system. This case provides valuable educational insights and may inform future device‐implantation strategies for Fabry disease and similar infiltrative cardiomyopathies.

## Author Contributions

**Hiroki Saito:** data curation, writing – original draft, investigation. **Yuya Suzuki:** conceptualization, writing – review and editing. **Shinichiro Yamada:** writing – review and editing, investigation, supervision. **Hideki Fujii:** resources, validation, writing – review and editing. **Kaoru Takami:** investigation, validation, resources. **Yu Takahashi:** formal analysis, visualization, validation. **Kimi Nishijyo:** investigation, resources. **Akihiro Yoshida:** supervision, writing – review and editing.

## Funding

The authors have nothing to report.

## Ethics Statement

Ethical approval was not required for this case report as it describes the clinical management of the patient and does not involve any experimental interventions. All clinical procedures were performed in accordance with institutional guidelines and the Declaration of Helsinki.

## Consent

Written informed consent was obtained from the patient for the publication of this case report and any accompanying images.

## Conflicts of Interest

H.F. has received lecture fees from Sumitomo Pharma, Takeda Pharmaceutical Company, Amicus Therapeutics, and Sanofi, and research support from JCR Pharma. All other authors declare no conflicts of interest.

## Data Availability

Data sharing is not applicable to this article as no datasets were generated or analyzed during the current study.
